# EBNA3C regulates p53 through induction of Aurora kinase B

**DOI:** 10.18632/oncotarget.3310

**Published:** 2015-01-21

**Authors:** Hem C. Jha, Karren Yang, Darine W. El-Naccache, Zhiguo Sun, Erle S. Robertson

**Affiliations:** ^1^ Department of Microbiology and the Tumor Virology Program, Abramson Cancer Center, Perelman School of Medicine at the University of Pennsylvania, Philadelphia, PA, United States of America

**Keywords:** EBV, Aurora Kinase B, p53, Phosphorylation, oncogenesis

## Abstract

In multicellular organisms p53 maintains genomic integrity through activation of DNA repair, and apoptosis. EBNA3C can down regulate p53 transcriptional activity. Aurora kinase (AK) B phosphorylates p53, which leads to degradation of p53. Aberrant expression of AK-B is a hallmark of numerous human cancers. Therefore changes in the activities of p53 due to AK-B and EBNA3C expression is important for understanding EBV-mediated cell transformation. Here we show that the activities of p53 and its homolog p73 are dysregulated in EBV infected primary cells which can contribute to increased cell transformation. Further, we showed that the ETS-1 binding site is crucial for EBNA3C-mediated up-regulation of AK-B transcription. Further, we determined the Ser 215 residue of p53 is critical for functional regulation by AK-B and EBNA3C and that the kinase domain of AK-B which includes amino acid residues 106, 111 and 205 was important for p53 regulation. AK-B with a mutation at residue 207 was functionally similar to wild type AK-B in terms of its kinase activities and knockdown of AK-B led to enhanced p73 expression independent of p53. This study explores an additional mechanism by which p53 is regulated by AK-B and EBNA3C contributing to EBV-induced B-cell transformation.

## INTRODUCTION

Epstein-Barr virus (EBV) is a γ-herpesvirus that establishes a lifetime infection in most of the adult human population [1]. Although infection is generally latent and asymptomatic, EBV can contribute to a number of lymphoid and epithelial malignancies in immunosuppressed individuals including post-transplant lymphoproliferative disorder, Burkitt's lymphoma, nasopharyngeal carcinoma, and other HIV-associated lymphomas [2]. In lymphoblastic cell lines, nine EBV proteins are latently expressed: six nuclear antigens (EBNA-1, -2, -3A, -3B, -3C, -LP), and three viral membrane proteins. EBNA2, EBNA3A, EBNA3C, EBNA-LP, and LMP1 are required for EBV-mediated transformation of primary B cells [3].

A growing number of studies have suggested an essential role for EBNA3C in viral associated-oncogenesis, specifically through direct interactions with regulator proteins responsible for maintaining cell-cycle checkpoints. Recently, we demonstrated that EBNA3C can block E2F1-mediated apoptosis induced by DNA damage, as well as facilitates the G1 to S transition by enhancing Cyclin D1 function [4, 5]. Furthermore, EBNA3C expression in cells is generally linked with chromosomal aberration, mitotic checkpoint defects, and aneuploidy [6, 7], and these effects of EBNA3C are typically achieved through the activation or repression of transcription. The N-terminus contains a domain that directly interacts with RBPJ/CSL, a transcription factor that interferes with Notch-mediated transcription [8]. EBNA3C can also associate with Nm23-H1, a metastasis suppressor protein and this activity can also regulate the tumor suppressor Necdin [9]. Other genes regulated by EBNA3C include BIM, FLNA, CD21, JAG1, NCALD, TCL1A, ITGA4, p16, IRF4, AK-B, and H2AX [10-16]. It has been shown that EBNA3C uses other transcription factors such as Gal4 and Sp1 to indirectly anchor itself to DNA and regulate gene expression [17]. HDAC1, CtBP, SMN, DP103, p300, prothymosin alpha, Sin3A, and NcoR are other transcription factors previously shown to interact with EBNA3C [9, 18-20].

One of the most important cellular proteins affected by EBNA3C is the p53 tumor suppressor. P53 maintains genomic integrity in multicellular organisms by activating DNA repair, arresting cells at the G1/S checkpoint, and inducing apoptosis [21]. Deletions or mutations of the TP53 gene are present in over half of human tumors [22]. P53 is activated in response to cellular stress, normally in the form of DNA or ribosomal damage, and can trigger different pathways to cell arrest or apoptosis [23]. The downstream signaling pathways of p53 involve a multitude of proteins in a complex network. We have shown that EBNA3C downregulates p53 transcription and binds to its transactivation domain, inhibiting p53-mediated transcriptional activity [24]. In this study, we investigated the important domains of p53 which are regulated by AK-B and EBNA3C to contribute to EBV-induced oncogenesis.

More recently, we discovered that EBNA3C up-regulates and stabilizes AK-B, a serine/threonine kinase that plays multiple roles which are integral to accurate cell division [15]. AK-B corrects errors in spindle-microtubule attachments and establishes chromosome bi-orientation in prometaphase [25]. Furthermore, depletion of AK-B disrupts the checkpoint and allows cells to prematurely enter anaphase [26]. During cytokinesis, AK-B targets proteins to the cleavage furrow for proper segregation of the cytoplasm to form two daughter cells [27]. Due to its numerous functions associated with critical cellular functions, aberrant AK-B expression is linked to many human cancers [27]. Extensive research has focused on the separate roles of p53 and AK-B in regulating numerous cellular activities. While this approach can provide an in-depth understanding of a single protein, it glosses over the numerous pathways that are linked to these two critical proteins and the regulatory activities that result from their interaction. AK-B can phosphorylate p53 at serine 215, which leads to polyubiquitination and degradation of the tumor suppressor [28]. P53 contains additional phosphorylation sites which include serine 315 which are susceptible to the activity of other aurora kinase AK-A, and may not be regulated by AK-B [29, 30]. Furthermore, enhanced AK-B expression is linked to p53 mutations in tumor cells. Therefore it is also possible that p53 may negatively regulate AK-B levels or its activities. We therefore investigated the interplay between these proteins to determine how these two proteins may regulate each other or maintain a fine balance important for metastasis. If p53 and AK-B regulates each other, it is likely that they co-exist in a finely tuned system under normal physiological conditions. However, an essential EBV latent protein like EBNA3C may have the potential to disrupt this equilibrium, and so lead to aberrant cell cycle and oncogenic activities which drives transformation of infected cells.

## RESULTS

### p53 and p73 expression are induced in EBNA3C knockout EBV infected PBMCs

**Fig 1:**
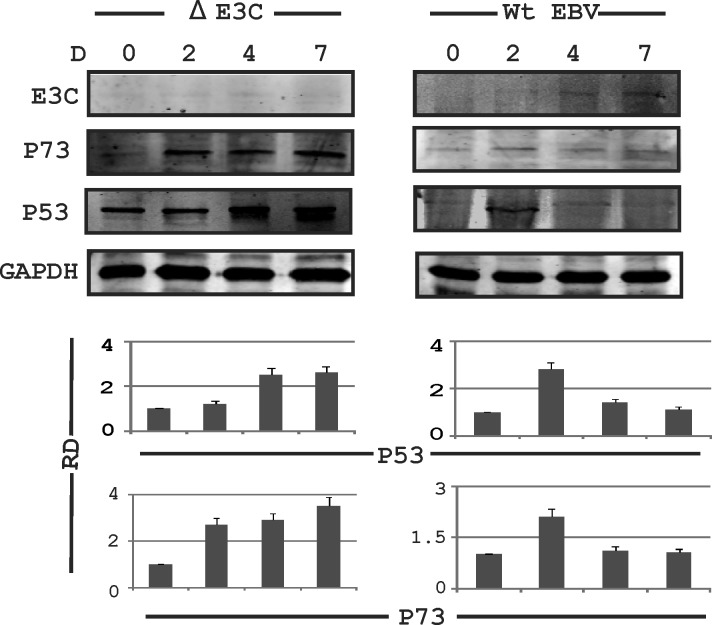
Expression of AK-B and p53 in EBV infected primary and stable cell lines PBMCs were subjected to the EBV wild type and delta EBNA3C EBV infection and blotted for EBNA3C, p53, p73 & GAPDH at 0, 2, 4 and 7 days. The delta EBNA3C studies showed an increase in level of p53 and p73 compared to WT EBV which showed a decrease. Here RD used for relative density for Western blot.

Previously, we had shown that EBNA3C up-regulates and stabilizes AK-B, leading to cell proliferation [15]. We found a consistent increase in AK-B expression in wild type EBV infected PBMCs 2 days after infection. This was demonstrated over time at 4 to 7 days. However, we saw weaker expression of AK-B in the initial days of infection when the EBNA3C knockout EBV virus was used to infect PBMCs [15]. AK-B is a known regulator of p53 [28]. Therefore we were interested in investigating the changes in the expression of this tumor suppressor after EBV infection. Interestingly, we observed that expression of p53 and its homolog p73 was enhanced at two days post infection but showed decreased expression at 4 days to 7 days post-infection with wild type EBV (Fig. 1, right panel). However, a consistent increase was found at the protein levels for both p53 and p73 in PBMCs infected with the delta EBNA3C knockout virus (Fig. 1, left panel). These results suggest that EBNA3C may have a role in regulating expression of these two tumor suppressors.

### EBNA3C regulates p53-mediated AK-B repression

**Fig 2:**
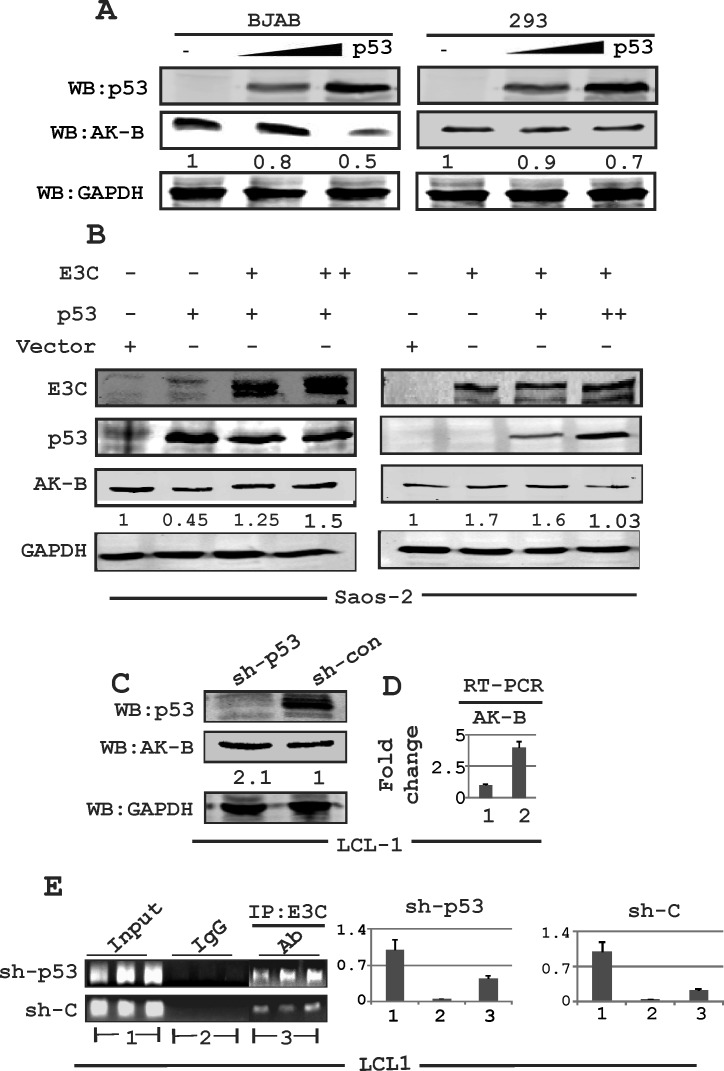
Expression of AK-B in EBNA3C positive and EBV transformed LCLs (A) Evaluating the levels of AK-B with dose dependent increase of p53 in BJAB and HEK-293T cells. (B) Evaluation of AK-B in a dose dependent manner in EBNA3C and p53 in Saos-2 (p53−/−) cells. (C, D) Determination of AK-B and EBNA3C at the protein and transcript levels in sh-p53 and sh-control in LCL1 stable cell line. Here 1 and 2 denoted to sh-control and sh-p53 in LCL1 cells. (E) In ChIP assay: stable sh-p53 and sh-control clones LCL1 cells were immunoprecipitated with A10 antibody, followed by real time PCR with AK-B ChIP primers. The amplified PCR products were fractionated on agarose gels. Here 1, 2 and 3 denoted to input, control IgG and IP:E3C group respectively.

To determine if p53 in return can directly contribute to down-regulation of AK-B, we transfected BJAB and HEK-293 cells with a p53 expression plasmid in a dose-dependent manner and performed Western blots, probing for AK-B protein levels (Fig. 2A). Endogenous AK-B levels were decreased with increasing levels of p53 in both cell lines, which was consistent with our expectations.

We previously showed that EBNA3C can bind to the AK-B promoter, and can directly up-regulate AK-B at both the transcription as well as protein levels [15]. To further investigate whether p53 could suppress or reverse the ability of EBNA3C to induce AK-B expression we transfected Saos-2 (p53−/−) cells with increasing amounts of p53 in a dose-dependent manner in the presence of EBNA3C and found that AK-B levels decreased to baseline levels (Fig. 2B). However, EBNA3C increased the expression of AK-B in the absence of p53 approximately 2-fold (Fig. 2B right panel, lane 2). In addition, transfecting p53 with increasing levels of EBNA3C showed that the levels of AK-B expression was gradually rescued from the inhibition by p53 as we increased the levels of EBNA3C (Fig. 2B, left panel). We also performed a knockdown assay using sh-p53 in LCL1 cells, in which the expression of AK-B was driven by only endogenous p53 and EBNA3C. Western blot and real-time PCR analysis revealed that AK-B levels were noticeably higher (2-3 fold) in p53 knocked down cells compared to vector control cells (Fig. 2C and 2D). Following these results, we speculated that p53 might have a dual role in suppressing the effects of EBNA3C on AK-B. Not only could it directly down-regulate transcription of AK-B, but it may also interfere with the association of EBNA3C with the AK-B promoter. We performed a chromatin immunoprecipitation (ChIP) assay in control and sh-p53 LCL1 cells using an antibody for endogenous EBNA3C (A10) [31]. The DNA samples were amplified by PCR with three sets of primers, each spanning a separate region of the AK-B promoter. The products were fractioned and quantified on an agarose gel. Our results confirm that the knockdown of p53 increased association of EBNA3C with the AK-B promoter region by approximately 2-3 fold (Fig. 2E).

### AK-B transcription regulated by p53 is modulated by EBNA3C

**Fig 3:**
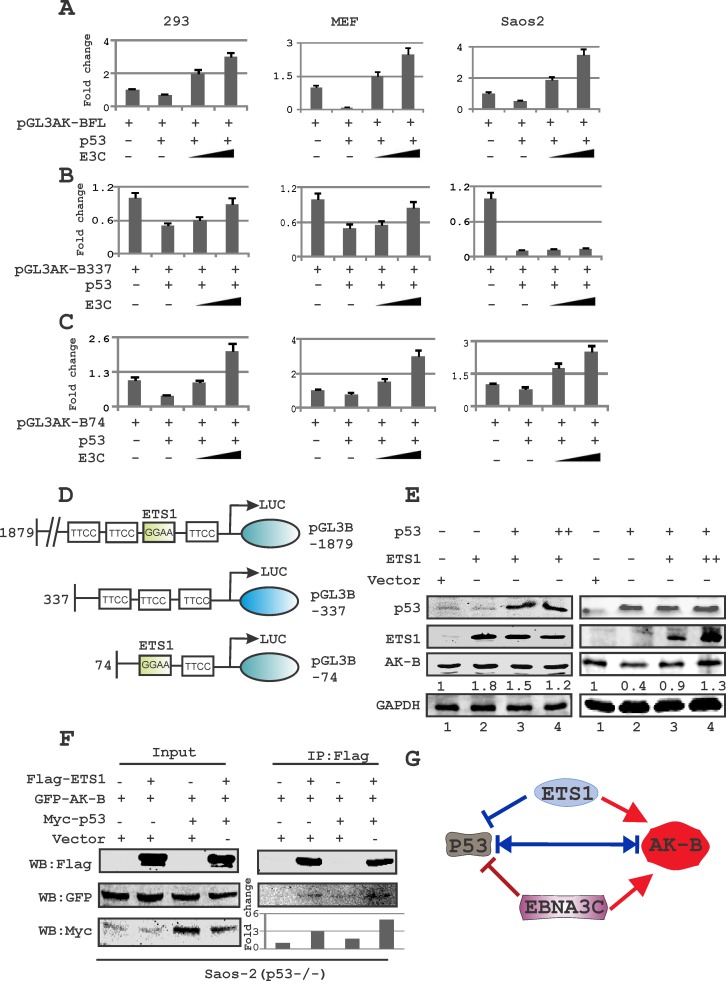
The ETS-1 motif in AK-B promoter is required for p53-mediated inhibition of AK-B (A-C) Reporter assays were performed in three cell lines: HEK-293T (p53+/+ & Mdm2+/+), Saos-2 (p53−/−), and MEF (p53−/− & Mdm2−/−). Cells were transfected with three plasmids – (A) AK-B-pGL3- full length (1879), (B) AK-B-pGL3-337, and (C) AK-B-pGL3-74, in a dose-increment manner with EBNA3C in the presence of the tumor suppressor p53. Graphs are plotted showing the relative luciferase unit (RLU/beta- gal activity). The pGL3 control plasmid was added to normalize transfection efficiency. The luciferase activity was measured as described in ‘Materials and Methods’ section. The mean values and standard deviations of three independent experiments are presented. 5% of the cell lysates were resolved by an SDS-PAGE gel to confirm that the transfection had been effective (data not shown). Rescue of AK-B promoter activity in presence of E3C is significant (p<0.05) with pGL3-AK-BFL and −74 compared to −337 promoter construct. (D) A representative picture of the AK-B wild type and truncated promoters are shown. (E) Evaluation of endogenous levels of AK-B in a dose dependent manner with EBNA3C and ETS-1 in Saos-2 (p53−/−) cells. (F) Immunoprecipitation of Flag-ETS-1 for AK-B in the absence and presence of p53 in Saos-2 (p53−/−) cells. (G) A representative picture for the equilibrium of AK-B, ETS-1, p53 and EBNA3C in cells.

If the level of p53 was maintained at a constant level with a dose-dependent increase in levels of EBNA3C, the levels of AK-B as determined by Western blot increased (Fig. 2B, left panel). As expected p53 alone decreased AK-B expression levels but this was rescued by increasing the levels of EBNA3C. Since p53 and EBNA3C have well-documented roles as transcription factors, we decided to investigate whether their regulation of AK-B occurred on the transcript level. We conducted luciferase assays using HEK-293, Saos-2 (p53−/−) and MEF (p53−/−,Mdm2−/−) cells to assess the promoter activity of AK-B after the transfection of p53, or p53 and EBNA3C, as well as control plasmids (Fig. 3A-C). The assay was repeated with two truncations of the AK-B promoter, one from −337 to −74 bp upstream of the transcription start site (TSS), and the other from −74 to the TSS [32]. Regardless of the promoter region used (Fig. 3D), the expression of p53 in this system markedly down-regulated AK-B promoter luciferase activity. Importantly, expression of EBNA3C in the presence of p53 restored and elevated AK-B transcription levels above those of control with the full length promoter as well as −74/+1 truncated promoter (Fig. 3A-C). However, we observed that EBNA3C was unable to achieve the same rescue effect with the −337/−74 truncated promoter region deleted for the ETS-1 binding site [32]. Additionally, the −337/74 promoter was dramatically suppressed when p53 was expressed in Saos-2 cells with no detectable change with EBNA3C (Fig. 3B).

### ETS-1 is required for hyper-activation of the AK-B promoter in the presence of p53

We speculated that the ETS-binding domain, located between −68 and −71bp upstream of the AK-B TSS, may be responsible for recruiting ETS-1 to the AK-B promoter region to modulate p53 activity. To determine whether ETS-1 attenuates the effect of p53 on AK-B, we transfected Saos-2 cells with a constant dose of ETS-1 with increasing amounts of p53 (Fig. 3E left panel). Western blot analysis showed that endogenous AK-B levels did not decrease. However, expression of ETS-1 can abrogate p53 repressor function. We therefore performed a similar experiment with the expression of p53 kept constant with increments of ETS-1. We observed that with ETS-1expression there was a moderate increase in AK-B protein levels which suggests that ETS-1 is important for p53 regulation of AK-B (Fig. 3E, right panel lanes 2-4). To determine if EBNA3C transactivation of the −337 truncated promoters could be restored by ETS-1, we performed luciferase assays with ETS-1 expressed in a dose-dependent manner. The results showed that ETS-1 expression rescued the inability of EBNA3C to reverse the negative effects of p53 on AK-B transcription (data not shown). Therefore ETS-1 may be able to contribute of AK-B transcription regulation through its association with the EBV transcription factor as well as by binding directly to its cognate sequences.

Wakahara et al previously reported that the ETS-1 binding domains in the AK-B promoter is critical for gene expression [32]. However, ETS-1 may have a transactivation function that is independent from its ability to oppose the p53 repressor activity [33]. Furthermore to determine if ETS-1 and AK-B can associate in a complex with and without p53, we performed immunoprecipitation in Saos-2 (p53−/−) cells with tagged ETS-1, AK-B and p53. Interestingly, the results demonstrated a greater association for AK-B with ETS-1 when p53 was present which suggests a critical role for p53 in a functional complex containing AK-B and ETS-1 (Fig. 3F).

### The P53 residues S215 and S315 are distinctively sensitive to AK-B and EBNA3C

**Fig 4:**
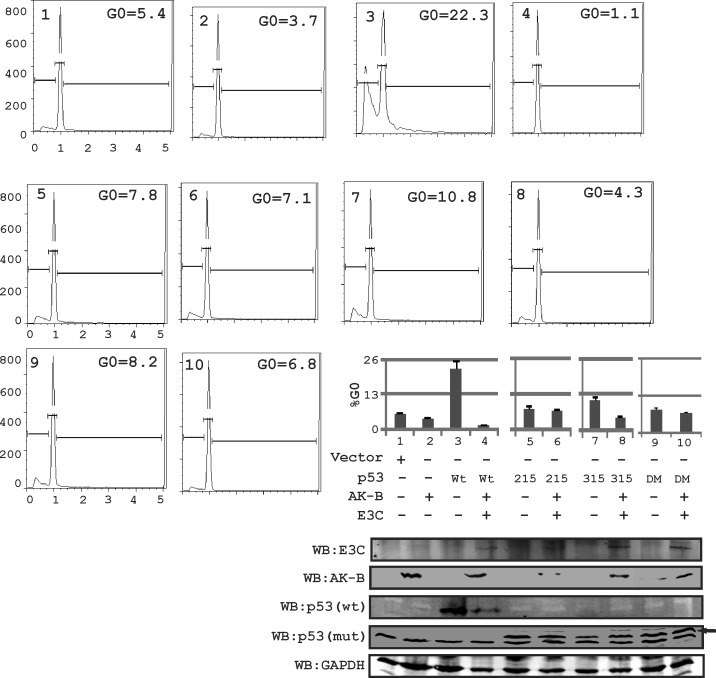
p53-induced apoptosis is regulated in the presence of AK-B and EBNA3C In MEF (p53−/−, Mdm2−/−) cells, using propidium iodide staining for determining the apoptotic or cell death population. Here the effect of wild type p53 and its mutants were evaluated on AK-B with the presence of EBNA3C. DM refer to double mutant of p53(residue 215 and 315 mutated). Here wild type p53 is myc tagged and mutant (mut) p53-215,315 and 215-315 are HA tagged. Black arrow represents p53 mutant bands probe with anti-HA antibody.

Uncontrolled growth may be caused by the impaired ability of cells to arrest in the G0 phase. To determine if phosphorylation of p53 at serine residues 215 and 315 is important for its regulation by AK-B and EBNA3C we mutated S215 and S315 to alanine and tested their ability to retain their tumor suppressor activities in the presence of EBNA3C and AK-B, which were monitored by apoptosis assays. Cells were transfected with plasmids and subjected to etoposide treatment to induce DNA damage. Using flow cytometry, we measured the percentage of cells that were successfully arrested in the G0 phase (Fig. 4). We found that cells expressing wild type p53 normally arrested with approximately 25% of cells similar to the p53 phospho-mutants. However, with the expression of EBNA3C and AK-B, the apoptotic effect of wild type p53 declined drastically when the wild type p53 was included. Importantly, the p53 S215A mutant lost greater than 50% of the wild type activity which did not change in the presence of AK-B and EBNA3C. Interestingly, the S315A p53 mutant was similar to wild type p53 but lost greater than 50% of the activity with EBNA3C and AK-B.

**Fig 5:**
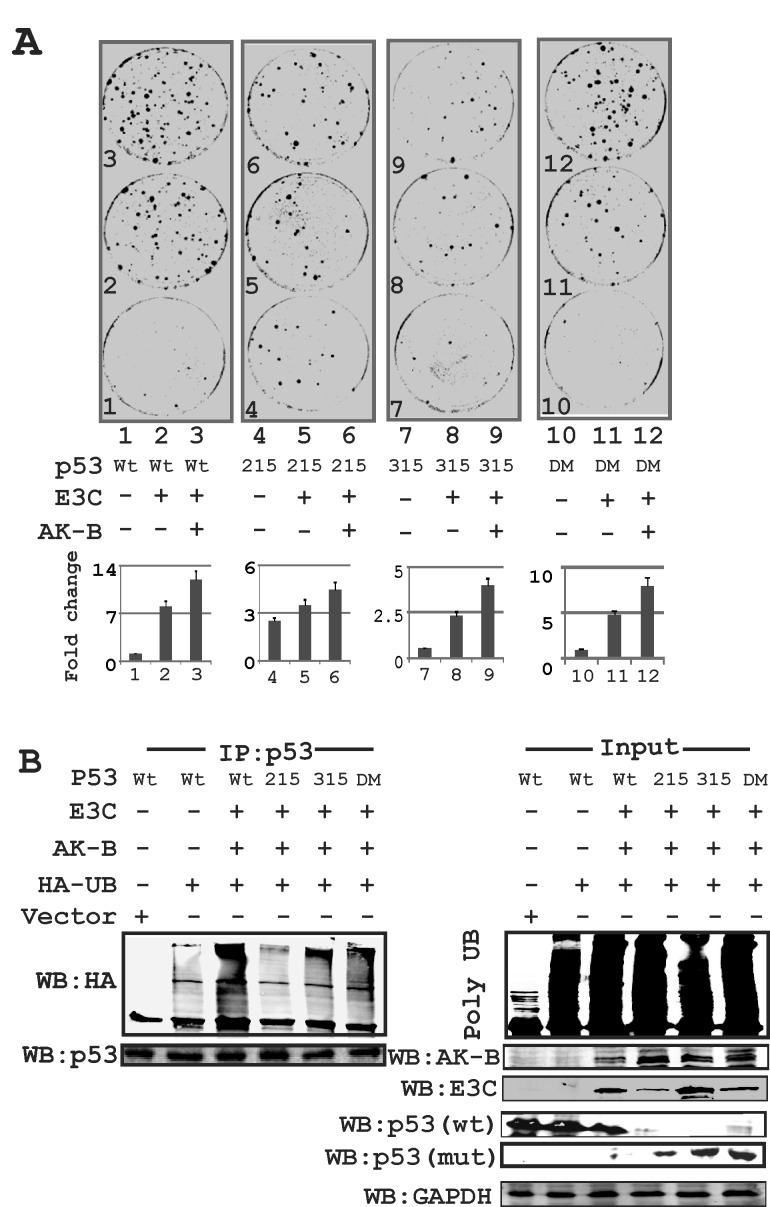
The p53 mutation at amino acid 215 is critical for AK-B and EBNA3C dependent cell proliferation (A) p53 wild type and mutants (215, 315, 215-315) in combination with AK-B and EBNA3C were evaluated by colony formation assay. Here DM refer to double mutant of p53(residue 215 and 315 mutated). (B) Ubiquitination assay- to monitor the ubiquitination status of endogenous p53 in response with AK-B and EBNA3C. We used p53 wild type and mutants (215, 315, 215-315) in this study. Immunoprecipitation was carried out with p53 antibody. Here DM refer to double mutant of p53(residue 215 and 315 mutated). Here wild type p53 is myc tagged and mutant (mut) p53-215, 315 and 215-315 are HA tagged.

Further we performed luciferase assays to compare the effect of wild type p53 and its phospho-mutants on AK-B promoter activity. Our results confirm that unlike the other p53 constructs, S215A was unable to suppress AK-B expression (data not shown) suggesting that the S215 residue may be an important contributor to the transcription activity of p53. To determine the effect of p53, EBNA3C, and AK-B on cell growth, we performed colony formation assay (CFA) using MEF cells expressing EBNA3C and AK-B in combination with wild type p53 and its phospho-mutants. Strikingly, we found that there was no significant increase in colony formation when EBNA3C and AK-B were expressed with the p53 S215 mutant (Fig. 5A). In contrast, wild type p53 by itself did not lead to substantial growth. However colony formation increased by more than ten-fold when AK-B and EBNA3C were expressed along with p53 (Fig. 5A). The P53 S215A mutant did not suppress growth completely, as the expression of this phospho-mutant in the absence of EBNA3C and AK-B showed an increase in colony formation by more than three-fold over wild type p53 (Fig. 5A). Therefore p53 S215A enhances colony formation but somewhat blunted the effects of EBNA3C and AK-B. The p53 S315A mutant also regulated cell growth albeit differently when compared to the p53 S215A mutant. We observed fewer colonies in the p53 S315A plates when compared to their corresponding wild type p53 regardless of whether AK-B and EBNA3C were expressed (Fig. 5A). The P53 S315A mutant was also slightly less responsive to the effects of AK-B and EBNA3C than the wild type. To determine the proliferative effects of AK-B alone, and EBNA3C domains on wild type p53, we performed CFA and observed that AK-B with the N-terminal truncation of EBNA3C can effectively nullify p53 tumor suppressor activity in MEF cells (data not shown). To further understand the functional regulation of p53 protein levels we performed ubiquitination assays and found that the p53 S215A mutant was not readily ubiquitinated when compared to wild type p53 or the S315 mutant (Fig. 5B). The p53 S315A mutant showed a ubiquitination pattern similar to the wild type p53 (Fig. 5B, compare lanes 4 with 3 and 5). The data from these experiments suggest that the p53 S215A can effectively suppress cell growth in the presence of AK-B and EBNA3C but is not readily polyubiquitinated and degraded. P53 S315 mutant seems to be less efficient at resisting ubiquitination and so suppress cell growth to a level that is less compared to the S215 A mutant or wild type p53.

### AK-B kinase activity is important for its stability which is regulated by EBNA3C

**Fig 6:**
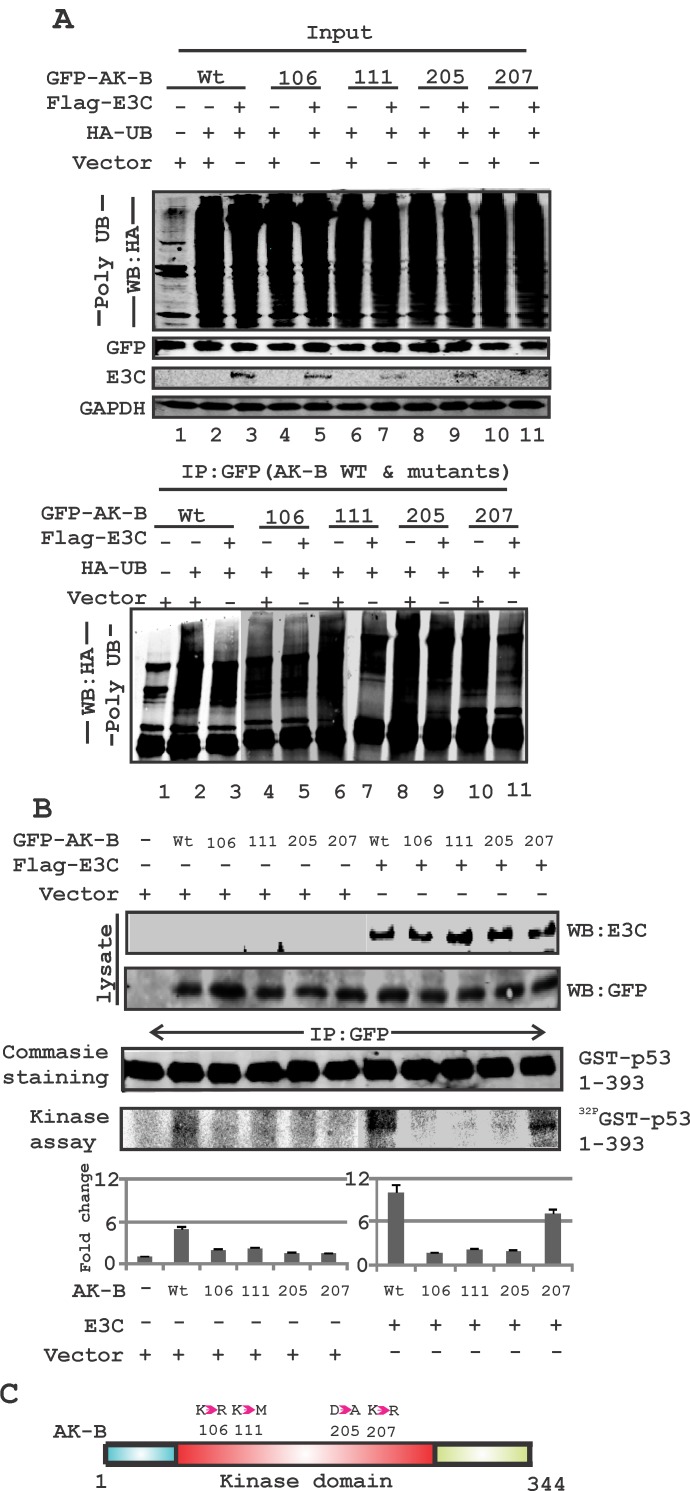
EBNA3C can regulate AK-B and p53 through their phosphorylation and ubiquitination (A) Determination of the ubiquitination status of wild type AK-B along with mutants (106, 111, 205 and 207 aa). Ubiquitination status was assessed with full length construct of EBNA3C for all AK-B constructs. (B) Kinase assay- wild type AK-B with mutations (106, 111, 205 and 207 aa) were evaluated with kinase assay in the absence/presence of EBNA3C in MEF cells. Immunoprecipitation was carried out with GFP antibody to immunoprecipitated the GFP-AK-B constructs. The full length p53-GST construct was used to determine the phosphorylation of p53. Blot were scanned and visualized using Typhoon PhosphorImager and quantified using the Image Quant software. (C) A schematic diagram illustrating - the mutations of Aurora kinase residues at positions 106, 111, 205 and 207.

AK-B is known to be degraded through targeted ubiquitination [34]. To determine the residues critical for EBNA3C-mediated stabilization of AK-B, we assayed the degree of ubiquitination for wild type AK-B and four mutants within the kinase domain, with and without EBNA3C. The difference between the two indicated the extent to which EBNA3C could suppress ubiquitination of the AK-B constructs (Fig. 6A, see lanes 2 & 3). We found that the K_106_D and D_205_A mutants were not effectively reduced, possibly by reduced ubiquitination or de-ubiquitination in the presence of EBNA3C. The wild type and K_207_R mutant showed much less ubiquitination in the presence of EBNA3C (Fig. 6A). The K_111_M mutant, whose kinase activity is shown to be greatly diminished but not completely dead, had a slightly lower level of ubiquitination with EBNA3C. The data therefore suggest that the kinase activity of AK-B is important for its stabilization by EBNA3C. To determine if EBNA3C-mediated reduction of AK-B ubiquitination directly impacts p53 phosphorylation, we utilized an *in vitro* kinase assay (Fig. 6B). We conducted the same transfections as in the ubiquitination assays, and cell lysates were incubated with the purified p53-GST substrate, and ^32^P labelled γ-ATP to facilitate the transfer of phosphate groups. In the absence of EBNA3C, we found that variations in p53 phosphorylation were barely discernible among the different AK-B mutant constructs, while wild type AK-B significantly phosphorylated p53 (Fig. 6B). However, when we performed the same experiment with the addition of EBNA3C, the differences were clearly seen. Wild type AK-B and its K_207_R mutant both showed reduced ubiquitination by EBNA3C and were able to phosphorylate p53 with an approximately 2-fold increase when compared to the kinase-dead mutants. Therefore, EBNA3C and AK-B specific residues that are important for its kinase activity both played a functional role in p53 phosphorylation, poly-ubiquitination and degradation. A schematic shows the position of the AK-B mutations within the kinase domain which were used in this study (Fig. 6C). These residues are likely to play a critical role in the stability of AK-B contributing to its kinase activity.

**Fig 7:**
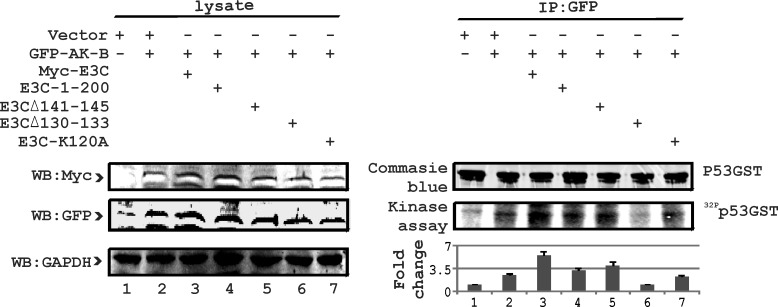
E3C residues 130-133 are critical for regulation of p53 phosphorylation To identify the specific residue of EBNA3C responsible for regulating the phosphorylation of p53 with AK-B, we used wild type EBNA3C, 1-200 aa acid and three other mutations (141-145, 130-133 and K120A). AK-B full length along with p53-GST full length was included in this study. 10 million MEF (p53−/−), (Mdm2−/−) cells were used in pull-down experiments for EBNA3C using myc (9E10) antibody. Empty vectors were added to normalize the transfection experiments. Blots were developed using the Typhoon Imager and quantified with Image Quant software.

To learn more about the regulation of p53 by EBNA3C, we performed *in vitro* kinase assays using p53-GST with wild type and mutant EBNA3C along with wild type AK-B. Earlier we established that residues 1-200 of EBNA3C were critical for regulation of p53 [24]. Furthermore, we determined that the binding of AK-B with EBNA3C was stronger within the 90-160 residues (15). To determine the specific residues of EBNA3C involved in the regulation of p53, we used specific point and deletion mutations of EBNA3C. These mutations of EBNA3C were reported to be critical for regulation of a number of cellular proteins [24]. Kinase assays were used to monitor phosphorylation of p53. We found that residues 130-133 of EBNA3C were most effective in contributing to p53 phosphorylation in the presence of AK-B (Fig. 7). This result corroborated our previous binding assays where binding of p53 and AK-B was strongest with residues 130-133 of EBNA3C. In addition, we sought to understand the functional implications of the reduced ubiquitination of AK-B. If AK-B were stabilized within the cell, its substrates will be phosphorylated to a greater extent leading to physiological change and disruption of homeostasis leading to cell proliferation.

### AK-B independently regulates p73 expression

**Figure 8:**
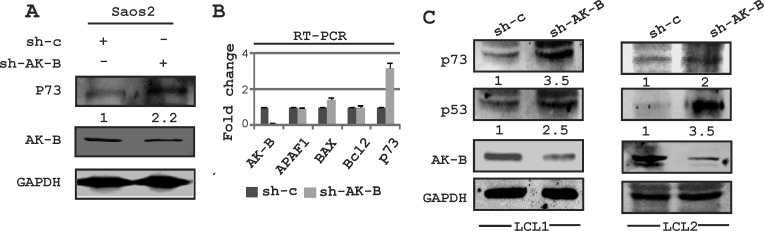
Expression of DNA damage markers in AK-B knockdown cells (A) Western blot analysis for the determination of p73 in AK-B knocked down Saos-2 cells compared to vector controls. GAPDH was used to evaluate the transfection and protein loading order. (B) Similarly transcript levels were determined for the DNA damage markers (p73, APAF1 and BAX) in AK-B knock down Saos-2 cells. The DNA repair marker (Bcl2) was also included. (C) Western blot analysis for the determination of DNA damage markers (p53 and p73) in AK-B knocked down LCLs were compared to vector controls.

**Figure 9:**
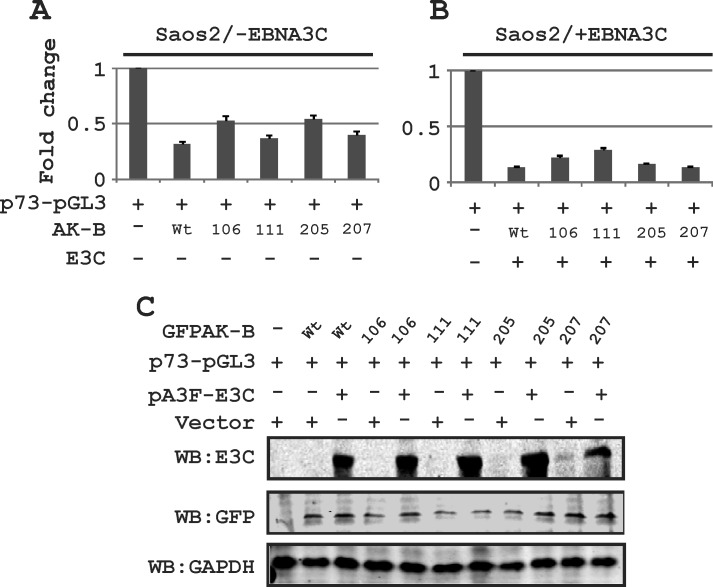
The effect of AK-B and EBNA3C on the p73 promoter activity (A) The p73 promoter was monitored in the presence of AK-B wild type and mutants (106, 111, 205, and 207). (B) The p73 promoter was investigated in the presence of AK-B wt, mutants and EBNA3C. (C) Western blots for transfections with different expression plasmids were performed to monitor expression levels.

In addition to p53, we wanted to determine which of the apoptotic pathway proteins were affected by AK-B. These include Bcl2, p73, APAF-1 and BAX1, the latter two of which are downstream proteins of p53. We transiently transfected Saos-2 (p53 −/−) cells with plasmids encoding short hairpin against AK-B to knock down the proto-oncogene. The transcript and protein levels were assessed by Western blot and RT-PCR analysis, respectively (Fig. 8A and B). We found that the levels of Bcl2, APAF-1, and Bax1 were not significantly different in AK-B knock down cells compared to the controls as monitored by transcript levels. However, p73 transcript and protein levels were dramatically increased in AK-B knockdown cells. This strongly suggests that AK-B may be directly involved in their downregulation. Further, we observed a strong up-regulation of p53 and p73 in AK-B knocked down LCL1 cells (Fig. 8C). To corroborate our findings at the transcript levels for p73, we added AK-B wild type and 4 mutants (aa 106, 111, 205 and 207) in the presence and absence of EBNA3C using the p73 promoter luciferase reporter (Fig. 9A and B). As anticipated, in the presence of AK-B wild type and its mutants the promoter activity of p73 was reduced greater than 2-fold (Fig. 9A). This reduction was further enhanced in the presence of EBNA3C to about 3-5 fold (Fig. 9B). Western blots to monitor expression of transfected plasmids are shown in Fig. 9C.

## DISCUSSION

AK-B and p53 are two crucial cellular regulators of the cell-cycle whose aberrant expressions are hallmarks of tumorigenesis. AK-B has multiple roles in mitosis such as the localization of centromeric proteins, the bi-orientation of chromosome attachments, and proper cytokinesis [35]. Over-expression of this kinase is commonly observed in numerous cancers including breast, pancreas, prostate, colon and others [26, 36-39]. p53 is a tumor suppressor that is inactivated in cancer either due to mutations in the TP53 gene or the repression of its gene expression by a host pro-tumorigenic factor as well as other activities [40]. Earlier studies from our lab and others reported that degradation of p53 mediated by EBV proteins are selective in targeting this tumor suppressor during viral infection and is important for oncogenesis [41, 42].

EBNA3C is a major viral-encoded transcription factor found in EBV-positive cells expressing the Type III Latency program. EBNA3C is essential for the transformation of B cells leading to proliferating lymphoblastoid cell lines. EBNA3C derails cell-cycle activities by interacting with multiple checkpoint proteins [5, 50, 51]. In the course of derailing the cell cycle checkpoint EBNA3C targets p53 as well as E2F1, pRB, BubR1, and H2AX [5, 43, 44].

Previous studies have linked EBNA3C, p53 and AK-B through multiple processes involved in gene expression, protein-protein interactions as well as post-translational modifications [24, 28, 45]. These studies demonstrated that EBNA3C down-regulated p53, and that AK-B is involved in a negative feedback loop with p53. These cell cycle regulators play important roles in controlling cell proliferation through the activities of the EBV latent EBNA proteins. In this study, we primarily explored the mechanisms by which EBNA3C induces expression of AK-B and suppresses p53 activity. Further experiments showed that these mechanisms can lead to the inhibition of apoptosis which drives the oncogenic process.

We have previously shown that EBNA3C can up-regulate and stabilize AK-B in stable cell lines as well as in EBV infected primary cells [15]. Further we also showed that knock down of EBNA3C during viral infection delayed the onset of AK-B expression in human PBMCs [15]. These results provide sufficient evidence showing that EBNA3C is able to directly regulate AK-B up-regulation during EBV infection. Moreover the expression of p53 in response to wild type EBV and the delta EBNA3C virus infection of primary cells appears to have increased by the second day in the knockout virus compared to a decrease with the wild type EBV virus. This result is similar to earlier studies from our group and others which showed higher expression of the p53 tumor suppressor on day 2 which led to a decrease in the presence of virus associated with infected primary B-cells [5, 46].

AK-B expression is induced in a number of tumors where p53 is defective [47]. In this study we show that p53 can directly down-regulate AK-B expression. AK-B is a proto-oncogene [48], which presents an important mechanism by which p53 suppresses oncogenesis. p53 is also able to down-regulate AK-B in LCL1 cells expressing EBNA3C and other EBV lytic genes [41]. This is significant because our interest in EBNA3C lies in its potential ability to distort the balance between AK-B and p53. We propose two mechanisms through which p53 regulates AK-B. Not only could it directly decreases AK-B promoter activity as supported by the luciferase assays, but it may also reduce the binding activity between EBNA3C and p53 which may lead to increased binding between EBNA3C and the AK-B promoter region.

While p53 can suppress AK-B in LCLs, the levels of EBNA3C present are much lower compared to that expressed in newly infected or reactivated cells. We sought to investigate whether increasing EBNA3C levels could overcome p53-mediated down-regulation of AK-B, tipping the balance between the two proteins. The results of our luciferase assays demonstrated that EBNA3C can overcome the effects of p53 to up-regulate AK-B. The truncation of the AK-B promoter (−337) without the ETS-1 binding domain failed to activate more than the control in response to EBNA3C, suggesting that an ETS-1 transcription factor is also required. ETS-1 factors are classified by their highly-conserved DNA-binding (ETS) domain and are involved in multiple facets of cellular regulation [49]. It has been reported that p53 and ETS-1 can form a complex *in vitro* and *in vivo* to regulate gene expression [50]. We propose that a similar mechanism is applicable to AK-B transcription. Additional luciferase assays with the −337 promoter showed that a combination of exogenously-expressed ETS-1 and EBNA3C was able to restore transcriptional activity suggesting that EBNA3C is directly involved in up-regulating AK-B likely through interaction with ETS-1.

We showed previously that EBNA3C is able to stabilize AK-B, and that this mechanism was dependent on the AK-B K_106_ residue [15]. Additionally, a similar kind of functional disruption was observed by another group [28]. The K_106_D mutant, which lacks the kinase activity, degraded more quickly in cyclohexamide stability assays and was not readily de-ubiquitinated by EBNA3C (15). We sought to determine if the kinase activity of AK-B was required for EBNA3C-mediated stability by comparing the behavior of the wild-type protein with that of several point mutants. Previously, Gully et al reported that the AK-B D_205_A mutant was essentially kinase-dead and that the K_111_M mutant displayed severely diminished kinase activity [28]. We selected these mutants with the anticipation that the K_111_M mutant would have stability between that of the wild type AK-B and its kinase-dead mutants. Additionally, a mutation at K_207_R, the residue required for AK-B SUMOylation gives rise to similar phenotypic defects as the D_205_A mutant [28]. Since the K_207_R mutant has uncompromised kinase activity, it was selected as a pseudo-control to compare with the D_205_A mutant. Our results revealed that the AK-B kinase activity is important for EBNA3C-mediated stabilization of p53, however K_207_R behave different than kinase mutants (106, 111, 205) as expected due to its pseudo-control nature of muation. Wild type AK-B and the K_207_R mutant were effectively stabilized through de-ubiquitination, but not the kinase dead mutants. Intriguingly, the changes in levels of kinase activity between the AK-B constructs were not particularly striking unless EBNA3C was present to stabilize those with active kinase domains. An earlier study from our group demonstrated that EBNA3C directly binds to p53 and inhibits its transcriptional activities [24, 45]. EBNA3C up-regulates AK-B, which can bind to p53 to form a complex mediated by the C-terminus of p53. To show the detrimental effects of this interaction, we conducted various functional assays using a combination of AK-B, EBNA3C and p53 wild type and mutant constructs. Gully et al previously showed that AK-B phosphorylates p53 at S215, with serve consequences to its function [28]. Phosphorylated p53 fails to activate the downstream genes p21 and pTEN because it is marked for degradation through the polyubiquitination-proteosome degradation pathway [51]. Furthermore, because AK-A can phosphorylate p53 at S315 leading to p53 polyubiquitination and degradation we postulated that S315 may similarly be susceptible to AK-B phosphorylation [29]. Prior to involving the S215A and S315A mutants in our experiments, we conducted a ubiquitination assay to confirm that the S215A and S315A mutants are less susceptible to ubiquitination than wild type p53. In the cell proliferation assay, we found that cells expressing mutant p53 did not proliferate as much as the wild type cells when AK-B and EBNA3C were co-expressed. Our rationale is that in cells expressing high levels of EBNA3C and AK-B, the p53 S215A and S315A mutants may not degrade as easily, thus retaining their tumor suppressor abilities and exerting control over cellular proliferation. This was reflected in our apoptosis assays where we found that the proportion of cells arrested in G0 in response to etoposide treatment decreased drastically when AK-B and EBNA3C were added to cells expressing wild type p53. However, less drastic declines were observed in cells expressing mutant p53. This data corroborated the work of Gully et al [28], and supports our hypothesis that p53 can be phosphorylated by AK-B at residue S315. Although resistance to poly-ubiquitination may explain why the p53 phospho-mutants were able to control cell proliferation in the presence of AK-B and EBNA3C, it does not explain why the S215A and S315A mutants had increased capability of suppressing cell proliferation when compared to wild type p53 under normal conditions. We speculated that the S215A mutation located in the DNA binding region of p53 may weaken the ability of p53 to activate or suppress downstream genes. This would explain why wild type p53 is normally more capable of arresting damaged cells than the S215A mutant. The reduced transcriptional activity of p53 S215A, combined with its resistance to ubiquitination and degradation, offers a potential explanation for its ability to buffer uncontrolled cell proliferation in the presence of EBNA3C.

**Figure 10:**
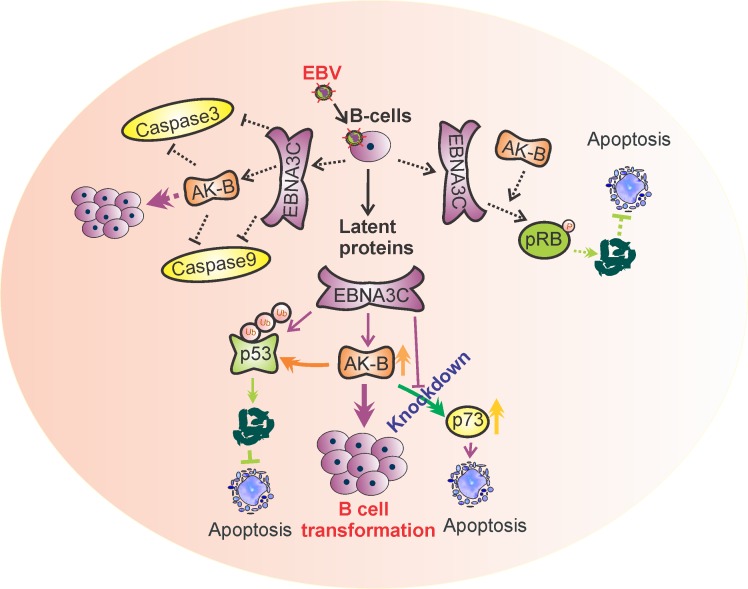
A model illustrating the association of AK-B, p53, p73 with EBNA3C This association drives progression of EBV-mediated B-cell transformation. Oncogenic activity of AK-B is accelerated in the presence of EBNA3C which targets the tumor suppressors p53 and p73. p53 is degraded through the ubiquitin mediated proteosome degradation pathway by AK-B and EBNA3C. p73 was inhibited synergistically through the activation of AK-B and EBNA3C at the transcript level. Inhibition of these tumor suppressors contributes to EBV-mediated B-cell transformation. The functional modulation of pRb and caspases through AK-B and E3C was published in earlier publication [15]..

Overall, we have provided strong evidence for a proposed mechanism whereby EBNA3C up-regulates AK-B which directly leads to p53 phosphorylation and degradation (Fig. 10). The evidence we have documented ties together numerous previous reports on the interplay between EBNA3C, AK-B and p53. P53-mediated apoptosis is one pathway that EBNA3C and AK-B can synergistically contribute to oncogenesis as well as others yet to be explored. In our studies, we used shRNA to silence the expression of AK-B and compared the transcript and protein levels of some common apoptotic markers. We found that the transcript and protein levels of p73 was up-regulated in the absence of AK-B. AK-B has the potential to regulate p73, which can lead to apoptosis independent of p53, while Bax1 enhancement was dependent on p53. In addition, we also demonstrated the effective inhibition of the p73 promoter by AK-B. This observation emphasizes the regulation of p73 by specific regions of AK-B. These results now revealed that similar to p53, the p73 tumor suppressor can also be directly targeted by the AK-B oncoprotein and serves as an important bridge linking the pro-apoptotic genes and oncogenic viral and cellular proteins important for driving EBV-infected cell proliferation.

## MATERIALS AND METHODS

### Ethics statement

The University of Pennsylvania CFAR Immunology Core provided human peripheral blood mononuclear cells (PBMCs). The donor gave written, IRB approved informed consent based on the declaration of Helsinki protocols. For our studies all samples were deidentified so that there were no links to any individual donor.

### Cell cultures, plasmids, antibodies, transfections and infection of human PBMCs

Wild type AK-B and the K_106_R mutant, cloned in pcDNA3/myc-his and GFP vectors, were gifts from Erich A. Nigg (Max-Planck Institute, Martinsried, Germany). The AK-B promoter regions, cloned in pGL3B vectors and used for luciferase assays, were gifts from Yukio Okano (Gifu University, Tsukasamachi, Japan). Aurora-B mutants cloned in GFP vector (Aurora BK111M, Aurora BD205A and Aurora BK207R) were kindly gifts from Marcos Malumbres (CNIO, Madrid, Spain).

The following cell lines were cultured in RPMI 1640 medium and other culture conditions described previously [15]. We used LCL1 and LCL2 (lymphoblastic cell lines); E3C7 and E3C10 (BJAB cells stably expressing EBNA3C clones); and BJAB (EBV negative). HEK-293, HEK-293T, Saos-2 and MEF cells were cultured in Dulbecco's modified Eagle's medium (DMEM) and other culture conditions described earlier [15].

The cloning of EBNA3C (full length) in the pEGFP, dsRed, pGEX2TK and pA3F and pA3M vectors, as well as various truncations in pA3F and pA3M (1–365, 366–620, 621–992), and pGEX2TK (90-365, 366-581, 582-792, 900-992, 90-129, 130-159, 160-190) vectors were previously described [45, 52]. Rabbit polyclonal antibodies specific for AK-B (H-75) and ubiquitin (FL-76) and mouse monoclonal antibodies against PARP1 (F-2) and GFP (F56-BA1) were obtained from Santa Cruz Biotechnology, Inc. (Santa Cruz, CA). Mouse monoclonal antibodies against GAPDH were purchased from US Biological Corp. (Swampscott, MA). Mouse monoclonal antibodies specific for the Myc epitope (9E10), Flag epitope (M2), and EBNA3C (A10) were produced as described earlier [4, 31]. Adherent cells and B-cells were transfected as described earlier [24]. PBMCs were obtained as described above in ethics section. All samples were deidentified with no links to the donor. We followed similar procedures as described earlier [15].

### RNA interference

Short-hairpin oligonucleotides were designed to knock-down EBNA3C, AK-B and p53 (Dharmacon Research, Chicago, IL) [4, 54]. The sequence for EBNA3C- CCATATACCGCAAGGAATA, AK-B-CGAGACCTATCGCCGCATC, control- TCTCGCTTGGGCGAGAGTAAG and p53- GACTCCAGTGGTAATCTA were used in Sh-RNA cloning. The single-stranded oligonucleotides were annealed and cloned into the Xho I and Mlu I restriction sites of the pGIPZ vector. As a control, an shRNA sequence that lacks complementary sequences in the human genome was also cloned into the pGIPZ vector (Dharmacon Research, Chicago, IL).

### Immunoprecipitation (IP) and Immunoblotting (IB)

Transiently transfected cells were harvested, washed with PBS, and lysed in RIPA buffer (10mM Tris, pH7.5, 150mM sodium chloride, 2mM EDTA, 1% NP40, protease inhibitor cocktail). Cell processing were performed as described earlier [41]. Bradford assays were used to standardize the protein samples within experiments, and 5% of the supernatant from each sample removed and used as input. The specific proteins were captured by rotating the lysates in fresh microcentrifuge tubes with 1 μg of antibody. Protein A/G beads were added to collect the immune complexes (rotation at 4°C). After two hours, the beads were pelleted and washed four times with ice-cold RIPA buffer.

For Western blot analysis, the input and IP complexes were fractionated by SDS-PAGE gel (8-12%) electrophoresis, and transferred to nitrocellulose membranes. The membranes were blocked in 5% milk in 1X-PBS for 40 minutes, washed three times in TBST buffer, and probed with the appropriate primary antibodies overnight at 4°C. Afterwards, the membranes were washed two times in TBST and incubated with appropriate infrared-tagged secondary antibodies at room temperature for 60 minutes. Scanning and densitometry analyses were performed by Odyssey Infra-Red scanner and software, respectively (Li-Cor Biosciences, Lincoln, NE).

### Luciferase reporter assay

Reporter assays were performed as described previously [55]. Briefly, plasmid mixes were prepared according to the specific experiment, and control plasmids were added as needed to normalize the amount of DNA. Following transient transfection, cells were incubated for 36 hours, lysed in reporter lysis buffer (Promega Inc., Madison, WI). Aliquots of the lysates were transferred to 96-well plates, an luciferase activity monitored as described earlier [4].

### Chromatin immunoprecipitation assay (ChIP)

ChIP assays were carried out as described previously [56]. LCL1 cells (10×10^6^) that were stably transfected with short-hairpin p53 or a control vector were cross-linked with formaldehyde, washed with 1X PBS, and lysed in cell lysis buffer (5mM Pipes, 85mM KCl, 0.5% NP-40). The nuclei were pelleted by centrifuging (5000 rpm for 5 mins). Next, nuclear lysis buffer (50mM Tris, 10mM EDTA, 1% SDS) was added to the nuclei and the DNA was sonicated for 2.5 minutes at 30-second intervals, producing fragments of approximately 600bp. The samples were pre-cleared with ssDNA/Protein A agarose beads (1 hours), and 20% was used as input. The remaining samples were each divided into two portions. One was rotated with the A10 antibody while the other was rotated with non-specific mouse serum, serving as the negative control. Both were kept at 4°C overnight. The DNA-protein-antibody complexes were collected by rotation with ssDNA/Protein A agarose beads (2 hours). The beads were collected, washed with a series of buffers, and treated with elution buffer (1% SDS, 0.1M NaHCO_3_). The mixture was shaken for 30 minutes. The samples were centrifuged (15000 rpm for 3 minutes) and the supernatant was transferred to a new tube. The input and ChIP samples were both incubated with RNase and NaCl (add to a final concentration of 0.3M) for reverse formaldehyde cross-linking. The DNA was purified, pelleted and dissolved in 30μl of water. Finally, the samples were amplified by PCR with three sets of primers, each spanning a separate region of the AK-B promoter. The products were run and quantified on agarose gels.

### Ubiquitination assay

20 million cells were transfected by electroporation with the plasmids for each experiment, including HA-tagged ubiquitin plasmids. Following 36 hours of incubation, the cells were treated with 20μM MG132 (Santa Cruz, Inc.) for another 12 hours to inhibit proteasome activity. The following procedures were as described earlier [4].

### Colony formation assay

Ten million MEF cells were transiently transfected with plasmids which include p53 wt and mutants, AK-B and EBNA3C by electroporation. After 36 hours of incubation, G418 was added to the cells to select for those containing plasmids. The media and antibiotic were replaced every other day for two weeks. Afterwards, cells were fixed on the plates with 3% PFA, stained with 0.1% crystal violet, and rinsed with water to remove excess dye. The plates were scanned using the LiCor-Odyssey system and growth was determined quantitatively using the Odyssey software.

### Apoptosis assay

Appropriate plasmids were transfected into MEF cells by electroporation. 24 hours post-transfection, the cells were incubated in 0.1% serum media for 12 hours and treated with 20 μM etoposide (MP Biomedicals) for six hours to induce DNA damage. The cells were then harvested for propidium iodide staining and flow cytometry analysis. Prior to PI staining, cells were fixed with an equal ratio of acetone : methanol for two hours at 4^o^C and washed with 1x PBS. The cells were treated with 50 μg/ml propidium iodide (Sigma, St. Louis, MO) and 1 μg/ml RNase (1 hour at 4^o^C). Flow cytometric analysis was conducted using the FACS Calibur cytometer (Becton Dickinson, San Jose, CA).

### GST protein purification and pull-down assay

GST-fusion proteins were purified essentially as described previously [57] with slight modifications. E. coli BL21-DE3 cells were transformed with GST-tagged p53 wild-type and mutant plasmids. The plasmids were induced with 1 mM IPTG and incubated overnight at 30^o^C. The cultures were pelleted by centrifuging (3000 RPM for 10 mins) and the pellet washed with STE buffer and resuspended in NETN buffer. Dithiothreitol (DTT) and 10% Sarkosyl in STE buffer were mixed into the solution, and the sample was sonicated to solubilized the proteins. The lysates were collected by centrifugation and the supernatant transferred and followed by addition of Triton X-100 in STE and Glutathione-Sepharose with rotation. The beads were collected by centrifugation (3000 rpm, 3 mins at 4°C) and washed with NETN buffer. 250μL of NETN was added to the washed beads, 20μL of which was resolved in an SDS-page gel and stained with coomassie blue to confirm expression of the fusion protein.

For GST pull-down assays, cells expressing AK-B was lysed using RIPA buffer. The lysates were pre-cleared with Glutathione-Sepharose beads, 10% was set aside as input, and the remainder was divided into two portions. One was rotated with the appropriate GST fusion protein-bound bead while the other was rotated overnight at 4^o^C with GST protein-bound beads to serve as a negative control.

### Quantitative PCR

Transiently transfected cells were harvested and washed with ice-cold PBS. RNA extraction was performed using the TRIzol reagent (Invitrogen), and 2.5μg of each RNA sample was converted to cDNA using the Superscript II Reverse Transcriptase Kit (Invitrogen Carlsbad, CA). Quantitative PCR was conducted using the following pair of primers. Primer sequence for AK-B forward- GGAGAGCTTAAAATTGCAGATTTTG, reverse- TGCAGCTCTTCTGCAGCTCCT, EBNA3C forward- AGAAGGGGAGCGTGTGTTGT, reversed- GGCTCGTTTTTGACGTCGGC, and GAPDH forward- CTCCTCTGACTTCAACAGCG, reversed- GCCAAATTCGTTGTCATACCAG. GAPDH amplification levels served as the internal control. Primer sequences of p73, Apaf1, p53, BAX, Bcl_2_ were previously reported [5, 41, 55]. The cDNA conversion and RT-PCR was performed as described earlier [57].

### Kinase assay

10 million cells were transfected with the indicated plasmids. Cells were harvested and Myc-tagged AK-B was immunoprecipitated (IP) using an antibody specific for the Myc epitope (9E10). A detailed protocol has been published earlier [15]. Radioactive proteins were resolved in SDS-PAGE, and band quantitation was performed using the Image Quant software (GE Healthcare Biosciences, Pittsburgh, PA).
